# Tolerance and Nature of Residual Refraction in Symmetric Power Space as Principal Lens Powers and Meridians Change

**DOI:** 10.1155/2014/492383

**Published:** 2014-11-12

**Authors:** Herven Abelman, Shirley Abelman

**Affiliations:** ^1^Biomedical Engineering Research Group, School of Electrical and Information Engineering, University of the Witwatersrand, Johannesburg, Private Bag 3, Wits 2050, South Africa; ^2^School of Computational and Applied Mathematics, University of the Witwatersrand, Johannesburg, Private Bag 3, Wits 2050, South Africa

## Abstract

Unacceptable principal powers in well-centred lenses may require a toric over-refraction which differs in nature from the one where correct powers have misplaced meridians. This paper calculates residual (over) refractions and their natures. The magnitude of the power of the over-refraction serves as a general, reliable, real scalar criterion for acceptance or tolerance of lenses whose surface relative curvatures change or whose meridians are rotated and cause powers to differ. Principal powers and meridians of lenses are analogous to eigenvalues and eigenvectors of symmetric matrices, which facilitates the calculation of powers and their residuals. Geometric paths in symmetric power space link intended refractive correction and these carefully chosen, undue refractive corrections. Principal meridians alone vary along an arc of a circle centred at the origin and corresponding powers vary autonomously along select diameters of that circle in symmetric power space. Depending on the path of the power change, residual lenses different from their prescription in principal powers and meridians are pure cross-cylindrical or spherocylindrical in nature. The location of residual power in symmetric dioptric power space and its optical cross-representation characterize the lens that must be added to the compensation to attain the power in the prescription.

## 1. Introduction

Perfectly centred lenses at the appropriate vertex distance may be manufactured or rotated inexactly compared to their specifications. The pursuit of perfect power and orientation for lenses would be wasteful. Imprecision in power and meridian location is acceptable to the human eye that is not a perfect detector of paraxial blur [1]. This paper presents a criterion as to whether residual power should be called for when principal powers of a lens differ from what is wanted or independently, associated meridians in a prescription are rotated around an optical axis [2]. Residues following surgery are not considered.

A lens with power to make up for the refractive error of an eye in every respect is called a prescription ophthalmic lens. A compensation lens is one that requires residual power, determined from spherocylindrical calculation, to make up entirely for the refractive error on the prescription for that eye. Initially compensation lenses have these changes: extreme powers are modified while their respective meridians stay put; then perpendicular meridians are allowed to rotate while powers are kept fixed. For modified bending (lens powers that vary with relative curvatures), a scalar (equivalent sphere) component and the antistigmatic (a Jackson cross-cylinder) power are present in the residual. For rotation, the scalar power component is zero so that the residual is a cross-cylinder lens that does not influence the position of the circle of least confusion. In existing work [3], bending along principal meridians as an eigenvalue modification was not considered; only fresh angles were adopted by the meridians of a compensation lens. Residual refraction also occurs, for example, when an eye, corrected in the spectacle plane, is unstable owing to cyclotorsion [4]. The development of the model precludes explicit consideration of orientation mechanisms of a contact lens and its fitting on the cornea and the tear lens. We determine the paraxial power to compensate for deviations in meridian directions and we use the identical fundamentals to correct departures in their principal powers for a lens.

**Figure 1:**
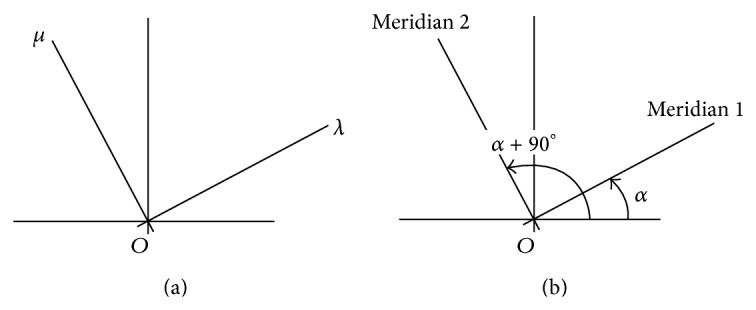
A manual lensometer measures meridians and corresponding powers on crosses. The lens centre is at the origin. Distinct lens principal powers *λ* and *μ* in (a) define eigenvalues of matrix **A** in (6) in columns of $\Lambda =\left(\begin{array}{cc} \lambda & 0\\ 0 & \mu \end{array}\right)$. These powers are along meridians at *α* and *α* + 90° in (b) that define eigenvectors of matrix **A** in (6) in the corresponding columns of $Q=\left(\begin{array}{cc} \cos\alpha & -\sin\alpha \\ \sin\alpha & \cos\alpha \end{array}\right)$ [6].

**Figure 2:**
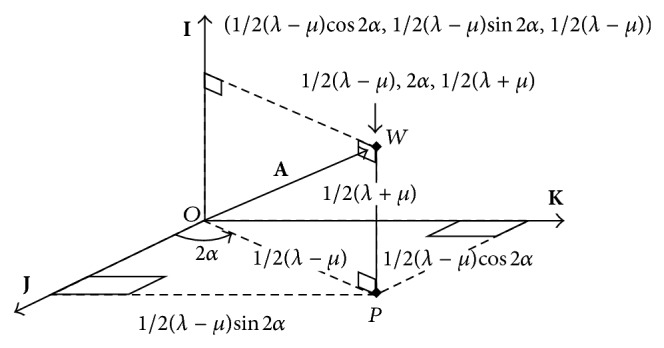
Power matrix **A** resolved into components along three orthogonal basic real matrices **I**, **J**, and **K** all with unit magnitude. The endpoint *W* of **A** is denoted by rectangular and cylindrical coordinates. Coordinates are expressed as lensometer measurements (some are arguments of trigonometric functions) that are identified with eigensystems of  **A**.

Principal power differences and principal meridians are seen to vary along perpendicular paths in symmetric power space. Data representing the lensometer readings or overrefractions in Figure 1(a) along the meridians on a cross as in Figure 1(b) are analogous to the output in the solution of an eigenvalue problem of some matrix [5, 6]. In addition to its representation as a measurement on an optical cross, a residual refraction power matrix can be plotted at a point in Figure 2. The nature of a residual refraction is identified from its location in symmetric dioptric power space.

Another measure, the magnitude of the residual lens power as a function of its sphere and cylinder for all axes, is determined and compared to the power of the residual cylinder, a mathematical quantity devoid of physical meaning. In this paper we determine the power and nature of the lens that complements the compensations to achieve the power in the prescription for various particular compensations in symmetric power space. Compensation on the eye affects the vision and, with the prescription, we determine the residual refraction and its nature in terms of orthodox measurements. Contrary to expectations, cylinder axis may change without power changing as a result; cylinder power may change without the axis changing as a result. Thus eigenvalue differences are dependent on a change in eigenvectors of a dioptric power matrix only if a patient cannot tolerate the blur and a practitioner chooses this dependence as a tolerance measure.

## 2. Method

Consider unit spherical lenses for which all meridians are equivalent and all unit cross-cylinder lenses with orthogonal meridians whose linear combinations are all components of most ophthalmic lenses. Their dimensionless basic matrices are

(1) $$\begin{array}{c} I=\left(\begin{array}{cc} 1 & 0\\ 0 & 1 \end{array}\right),\;\;J=\left(\begin{array}{cc} 1 & 0\\ 0 & -1 \end{array}\right),\\ K=\left(\begin{array}{cc} 0 & 1\\ 1 & 0 \end{array}\right),\;\;L=\left(\begin{array}{cc} 0 & -1\\ 1 & 0 \end{array}\right) \end{array}$$

with **I**, **J**, and **K** being symmetric [7]. Dimensions are attached to their coefficients in a linear sum. We multiply corresponding entries of, say, **I** and **I**, add the products, and divide the sum by 2. This produces a scalar product 〈**I**, **I**〉 of this matrix pair [5, 8, 9]. It is easy to verify that scalar products for every ordered pair of basic matrices are

(2) $$\begin{array}{c} \left(\begin{array}{cccc} \langle I,I\rangle & \langle I,J\rangle & \langle I,K\rangle & \langle I,L\rangle \\ \langle J,I\rangle & \langle J,J\rangle & \langle J,K\rangle & \langle J,L\rangle \\ \langle K,I\rangle & \langle K,J\rangle & \langle K,K\rangle & \langle K,L\rangle \\ \langle L,I\rangle & \langle L,J\rangle & \langle L,K\rangle & \langle L,L\rangle \end{array}\right)=\left(\begin{array}{cccc} 1 & 0 & 0 & 0\\ 0 & 1 & 0 & 0\\ 0 & 0 & 1 & 0\\ 0 & 0 & 0 & 1 \end{array}\right). \end{array}$$

In (2), positive square roots of entries 〈**I**, **I**〉, 〈**J**, **J**〉, 〈**K**, **K**〉, and 〈**L**, **L**〉 indicate that these matrices are of unit magnitude. Off-diagonal entries are the numerators in the cosines of the angles between each matrix pair [5, 8]. Since these cosines are all zero, all distinct matrices in any order in the brackets 〈*o*, Δ 〉 are orthogonal. Later we represent the mutual orthogonality of **I**, **J**, and **K** by plotting them along three perpendicular directions. The orthogonality of **I**, **J**, **K**, and **L** justifies known calculations with their coefficients that carry the magnitude and dimensions and spreadsheet use.

Consider any lens prescription with principal powers and meridians *λ* along *α* and *μ* along *α* + 90° on an optical cross. In Figure 1(a), nonzero powers *λ*, *μ* are scalars formatted as eigenvalue entries [6] in the matrix [7]

(3) $$\begin{array}{c} \Lambda =\left(\begin{array}{cc} \lambda & 0\\ 0 & \mu \end{array}\right)=\frac{\lambda +\mu }{2}I+\frac{\lambda -\mu }{2}J \end{array}$$

and, along lines at polar angles *α* and *α* + 90° in Figure 1(b), corresponding meridians are formatted as vectors and column entries in the matrix

(4) $$\begin{array}{c} Q=\left(\begin{array}{cc} \cos\alpha & -\sin\alpha \\ \sin\alpha & \cos\alpha \end{array}\right)=\cos\alpha I+\sin\alpha L \end{array}$$

of eigenvectors [6] of matrix **A** whose structure follows in (6).

Scalar products of matrices determine coefficients of **I**, **J**, **K**, and **L** in the following equations. In (3) the scalar product 〈Λ, **I**〉 is the coefficient in dioptres of **I** and the coefficient in dioptres of **J** is 〈Λ, **J**〉. Scalar products of Λ with **K** and **L** are the zero coefficients of **K** and **L**. In (4) the scalar product 〈**Q**, **I**〉 is the dimensionless coefficient of  **I**, coefficients of **J** and **K** are 〈**Q**, **J**〉 = 0 = 〈**Q**, **K**〉, and 〈**Q**, **L**〉 is the dimensionless coefficient of **L**.

Lensometer measurements *λ*, *μ*, and *α* become matrix entries in (3) and (4). As matrices of eigenvalues and eigenvectors, these matrices are multiplied [6] (not scalar products)

(5) QΛQ−1=cos⁡αI+sinαL×λ+μ2I+λ−μ2Jcos⁡αI+sinαL−1

to give the symmetric (independent of **L**) dioptric power matrix

(6) $$\begin{array}{c} A=\frac{1}{2}\left(\lambda +\mu \right)I+\frac{1}{2}\left(\lambda -\mu \right)\cos2\alpha J+\frac{1}{2}\left(\lambda -\mu \right)\sin2\alpha K \end{array}$$

as a linear combination. The units of **A** and those of the coefficients **I**, **J**, and **K** are dioptres. Equations (2) and (6) are fundamental equations for this paper.

Orthogonality of **I**, **J**, and **K** allows one to place unit matrices along three perpendicular directions in Figure 2 and represent **A** in (6) by its unique components called the scalar coordinate on the **I**-axis, the ortho-antistigmatic coordinate on the **J**-axis, and the oblique antistigmatic coordinate on the **K**-axis, respectively.

From (2), the scalar product

(7) A,A︸OW2=12λ+μ2I,I+12λ−μcos⁡2α2J,J +12λ−μsin2α2K,K=12λ+μ2︸PW2 +12λ−μcos⁡2α2+12λ−μsin2α2.︸OP2

We notice that the sum of the squares of the coefficients in (6) is the square of *OW* in D^2^ in Figure 2 by Pythagoras' theorem. The strength or magnitude of the power **A** is then the length *OW* in dioptres. Strength or magnitude ‖**A**‖ is significant for residual powers and is independent of meridian angles in (4):

(8) $$\begin{array}{c} \left\|A\right\|=\sqrt{\langle A,A\rangle }=\frac{\sqrt{2}}{2}\sqrt{\lambda^{2}+\mu^{2}}. \end{array}$$

Point *W* in Figure 2 is also denoted by cylindrical coordinates ((*λ* − *μ*)/2,2*α*, (*λ* + *μ*)/2). Equivalent sphere (scalar) power is (*λ* + *μ*)/2 and the cross-cylinder has power (*λ* − *μ*)/2 along a meridian *α*.

**Figure 3:**
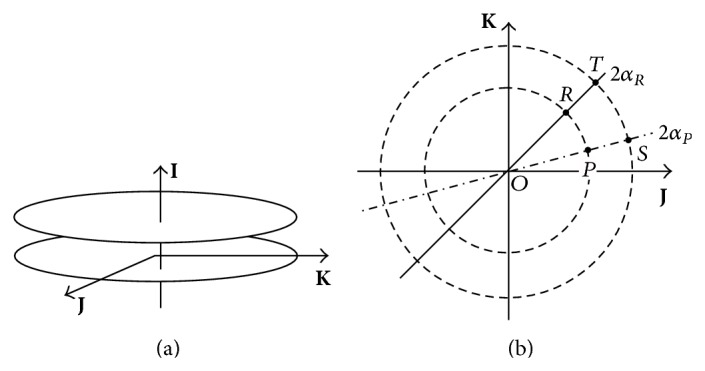
In (a) members of a family of planes are perpendicular to the axis **I** of scalar (stigmatic) powers. A particular plane through the origin contains powers called antistigmatic in (b) where the **I** of scalar powers emerges from the page at *O*. The prescription at *P* in Figure 2 is represented as polar coordinates ((*λ* − *μ*)/2, 2*α*) and as rectangular coordinates (1/2(*λ* − *μ*)cos⁡2*α*, 1/2(*λ* − *μ*)sin2*α*) in the plane in (b). Along the paths, these quantities that characterize arrays vary individually (from *P* to *S* and *P* to *R*) or in combination (from *P* to *T*).

In Figure 3(a) a family of planes perpendicular to **I** include powers that satisfy (*λ* + *μ*)/2 = constant which is the semitrace of the dioptric power matrix. Where this constant is zero, this plane contains the axes **J** and **K** and the lens power **A**, with zero trace, is a pure cross-cylinder lens: principal powers that are equal and opposite, called antistigmatic [10, 11], are located in the plane (*λ* + *μ*) = 0 and in Figure 3(b) where diameters intersect their circles at, say, *λ* and −*λ* on opposite sides of the origin *O*. On a lens, powers *λ* and −*λ* are along perpendicular meridians. Matrix **I** emerges from the page at *O*. In Figures 2 and 3(b) point *P* in the plane is identified by its polar coordinates ((*λ* − *μ*)/2,2*α*) [10, 12].

Equations (3) and (4) contain the basic variables to consider bending excess and rotation of a lens, respectively, relative to a given prescription in symmetric power space. A general result for any polar coordinates is

(9) $$\begin{array}{c} \left(\frac{\left(\lambda -\mu \right)}{2},2\alpha \right)=\left(-\frac{\left(\lambda -\mu \right)}{2},2\left(\alpha \pm 90^{{}^{\circ}}\right)\right). \end{array}$$

Particular to ophthalmic lenses, the sign of the cylinder semipower has changed and the meridian has been moved by 90° on the right, known as cylindrical transposition. Their equality, however, implies that powers represented by transposed lens coordinates and the given power in its coordinates plot at identical points in the antistigmatic plane.

Rectangular coordinates [10, 12] (1/2(*λ* − *μ*)cos⁡2*α*, 1/2(*λ* − *μ*)sin2*α*) may represent the intersection at a point like *P* in Figure 3(b) of each circle in a family of circles centred at the origin and their diameter of length (*λ* − *μ*) in the direction of [cos⁡2*α*, sin2*α*]^*T*^, a column vector. Eigenvectors of **A** in (4) vary on a circumference of a circle in Figure 3(b) which is the path for constant differences in eigenvalues (cylinder power, (*λ* − *μ*)) of the dioptric power matrix. Differences in the eigenvalues (*λ* − *μ*), seen in (6) for **A**, vary along circle diameters in Figure 3(b) which are the paths where the eigenvectors along the principal meridians are constant in direction. Powers of a prescription and a compensation lens are each represented by the coordinates at the intersection of the radial and circular paths.

Given that the lens prescription (represented at point *P* in Figure 3(b)) is *λ*_*P*_ along *α*_*P*_ and *μ*_*P*_ along *α*_*P*_ + 90°, then, from (6), the power of a lens that fully compensates the refractive status of an astigmatic eye without cyclotorsion [4] is

(10) AP=12(λP+μP)I+12λP−μPcos⁡2αPJ+12λP−μPsin2αPK.

Let the power of the lens change to point *S* in Figure 3(b) by curving it differently along principal meridians. If the compensation is *λ*_*S*_ along *α*_*P*_ and *μ*_*S*_ along *α*_*P*_ + 90°, then, from (6), the power of a differently curved lens is

(11) AS=12(λS+μS)I+12λS−μScos⁡2αPJ+12λS−μSsin2αPK.

These changes could result from prescription lenses changing in curvature along the given meridians on the cornea. Rotate the prescription lens *P* fixing existing curvature to point *R* where the compensation lens has principal meridians along *α*_*R*_ in Figure 3(b), *λ*_*P*_ along *α*_*R*_ and *μ*_*P*_ along *α*_*R*_ + 90°. Then

(12) AR=12(λP+μP)I+12λP−μPcos⁡2αRJ+12λP−μPsin2αRK.

Prescription *P* moves along the arc in Figure 3(b) and the compensation at *R* results. A numerical example where principal powers and their meridians in the compensation at point *T*, *λ*_*T*_ along *α*_*R*_ and *μ*_*T*_ along *α*_*R*_ + 90°, differ simultaneously from those in the prescription lens at *P* is considered. This and lens power compensation options in (11) and (12) need to be augmented to make up the prescription in (10). Numerical values of the coefficients of **I**, **J**, and **K** in (6)–(10) could be placed in columns headed, say, **I**, **J**, and **K**. However **I**, **J**, and **K** are independent basic matrices of standard lenses or surfaces and their coefficients indicate how much power each one contributes to the power matrix for an ophthalmic lens.

The power **G**_1_ that must be added to **A**_*S*_ to give **A**_*P*_ is called the residual power

(13) $$\begin{array}{c} G_{1}=A_{P}-A_{S}. \end{array}$$

That is,

(14) G1=12(λP−λS+μP−μS)I+12λP−λS−μP+μScos⁡2αPJ+12λP−λS−μP+μSsin2αPK.

In Figure 3(b), eigenvalues of **A**_*P*_ have become those of **A**_*S*_ in (13) while their eigenvectors are identical. This means that principal meridians on the compensation *S* are those on the prescription *P*. As the lens bends on the cornea and changes shape along the given meridians, a change in cross-cylinder power (in the coefficients of **J** and **K** in (14)) occurs in a patient's compensation and in the antistigmatic plane of Figure 3(b). Radial changes of power in this plane modify the scalar power (coefficient of  **I**, (14)). A practitioner adjusts this (spherical) power as the cylinder power goes from *P* to *S* in Figure 3(b) in order to keep the position of the circle of least confusion invariant in the eye. The symmetric residual power matrix **G**_1_ has the eigenfactors [6]

(15) $$\begin{array}{c} \Lambda =\left(\begin{array}{cc} \lambda_{P}-\lambda_{S} & 0\\ 0 & \mu_{P}-\mu_{S} \end{array}\right),\\ Q_{P}=\left(\begin{array}{cc} \cos\alpha_{P} & \cos\left(90^{{}^{\circ}}+\alpha_{P}\right)\\ \sin\alpha_{P} & \sin\left(90^{{}^{\circ}}+\alpha_{P}\right) \end{array}\right) \end{array}$$

that, as in Figure 1, can be represented on a power cross where the principal powers are *λ*_*P*_ − *λ*_*S*_ along *α*_*P*_ and *μ*_*P*_ − *μ*_*S*_ along 90° + *α*_*P*_. The strength or magnitude of the residual power **G**_1_ is obtained from the eigenfactor Λ of **G**_1_ and (8):

(16) $$\begin{array}{c} \left\|G_{1}\right\|=\frac{\sqrt{2}}{2}\sqrt{{\left(\lambda_{P}-\lambda_{S}\right)}^{2}+{\left(\mu_{P}-\mu_{S}\right)}^{2}}. \end{array}$$

This square root of the scalar product 〈**G**_1_, **G**_1_〉 [8] is independent of the meridians in **Q**_*P*_. Units of ‖**G**_1_‖ are dioptres and we may choose a small value for this scalar, say, 0.12 D, such that the magnitude of the residual power ‖**G**_1_‖ due to bending change of the lens should not exceed 0.12 D for principal powers *λ*_*P*_, *λ*_*S*_ and *μ*_*P*_, *μ*_*S*_ along any perpendicular meridians that coincide in the prescription and compensation lenses.

Suppose the prescription at *P* moves along the arc in Figure 3(b) to the compensation at *R*. Does this path also introduce a residual power along the axis **I**? The prescription at *P* has a power matrix given by (12). The residual power,

(17) G2=AP−AR=−(λP−μP)sin⁡(αP−αR)×{sinαP+αRJ−cos⁡(αP+αR)K},

follows by subtracting rows in corresponding columns. We find **G**_2_ with a zero scalar component of dioptric power in the antistigmatic plane of Figure 3(b) where powers have zero trace. Nonzero functions of principal meridians and powers are entries of a symmetric **G**_2_ in (17) having equal and opposite residual powers along perpendicular meridians. Suppose the principal power *λ*_2_ is along a meridian at an angle *θ* and *μ*_2_ = −*λ*_2_ is the power along 90° + *θ*. In these terms (6) becomes

(18) $$\begin{array}{c} G_{2}=\lambda_{2}\left(\cos2\theta J+\sin2\theta K\right). \end{array}$$

**Figure 4:**
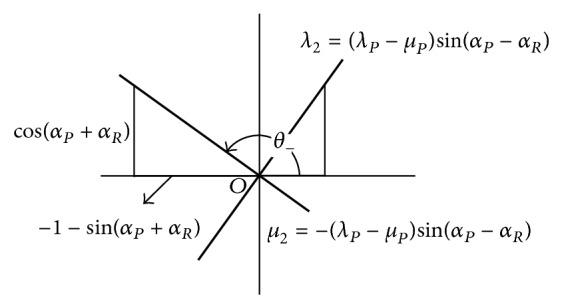
Coordinates of meridians. Principal powers *λ*_2_, *μ*_2_ = −*λ*_2_ of a cross-cylinder lens and perpendicular meridians *θ* for power matrix **G**_2_ on an optical cross that are added to the lens compensation at *R* whose eigenvectors differ from those of the given prescription at *P* in Figure 3(b) where eigenvalues of **G**_2_ remain unchanged on an arc *P* to *R*.

Coefficients of **J** and **K** in (17) and (18) are equated to yield the solution for the eigenpowers *λ*_2_, *μ*_2_ along meridians *θ* in Figure 4, for matrix **G**_2_. The residual cross-cylinder lens powers are

(19) λ2=(λP−μP)sin(αP−αR)along  a  meridian  at  θ+=αP+αR+90°2,μ2=−(λP−μP)sin(αP−αR)along  a  meridian  at  θ−=αP+αR−90°2.

The meridian of the prescription at *P* is advanced to *R* in Figure 3(b). Misalignment (*α*_*P*_ − *α*_*R*_) of the wanted meridian *α*_*P*_ causes a residual lens meridian to straddle the mean angle of the meridians at *P* (wanted) and *R* (supplied) by 45°. We have confirmed that, as meridians in a lens prescription are rotated, the compensation should be augmented by a pure cross-cylinder in (17) and (19). The residual cross-cylinder power at *R* is represented schematically in Figure 4 as principal meridians in terms of components of an eigenvector, angles and corresponding powers.

A measure of how far apart the lens prescription is from the compensation is the dioptric strength or magnitude of the residual power **G**_2_ in (17) that is a function of its sphere and cylinder. As in (8), to obtain ‖**G**_2_‖, we square *λ*_2_ and *μ*_2_ in (19), add them, and take the positive square root:

(20) $$\begin{array}{c} \left\|G_{2}\right\|=\left|\left(\lambda_{P}-\mu_{P}\right)\sin\left(\alpha_{P}-\alpha_{R}\right)\right|. \end{array}$$

Units of ‖**G**_2_‖ are dioptres and are independent of the meridians in (19). We choose a small value such that the magnitude of the residual power ‖**G**_2_‖ due to rotation of the principal meridians of the lens from *α*_*P*_ to *α*_*R*_ should not exceed 0.12 D for a cylinder of power *λ*_*P*_ − *μ*_*P*_. Consider

(21) $$\begin{array}{c} |\left(\lambda_{P}-\mu_{P}\right)\sin\left(\alpha_{P}-\alpha_{R}\right)|\leq 0.12\;\text{D}. \end{array}$$

Suppose we express the cross-cylinder residual power in Figure 4 as sphere, cylinder, and axis and only the cylinder component whose magnitude is

(22) $$\begin{array}{c} C≔\left|-2\left(\lambda_{P}-\mu_{P}\right)\sin\left(\alpha_{P}-\alpha_{R}\right)\right| \end{array}$$

is selected and attributed to the principal meridian misalignment. It may also serve as a tolerance measure not to exceed, say, 0.12 D. This result is used [13] but its effect is considered. If such a cylinder were added to a prescription, one would need to change the sphere so that, in effect, a cross-cylinder lens is added to keep the circle of least confusion on the retina. In other words, residual cross-cylinder lens power should not affect the equivalent sphere regardless of the original or resultant cylinder power. While the residual cylinder (devoid of residual sphere) is actually double the dioptric strength (see (20)) or magnitude ‖**G**_2_‖ of the residual power and has identical variables, residual cylinder lacks the effect of a cylinder in a prescription. The result for *C* is but a recipe used to estimate a tolerance [13] that seems devoid of physical meaning on its own. Previous (pre-2005) tolerance tables (cross-cylinder power attributed to meridian misalignment) differ by a factor 2 from the present-day tables (induced “cylinder” power attributed to meridian misalignment).

## 3. Examples

Two numerical examples illustrate the concepts discussed.

**Example 1 .**

A lens has the required cylinder power 2.00 D but the prescribed axis in the compensation is advanced by 5°. Estimate the residual cylinder power.

*Solution*. We have been given processed information: *λ* − *μ* = 2 D and *α* − *α*′ = 5°. We are thus only able to write (17) as

(23) $$\begin{array}{c} G_{2}=-2\sin5^{{}^{\circ}}\left\{\sin\left(\alpha +\alpha^{\prime }\right)J-\cos\left(\alpha +\alpha^{\prime }\right)K\right\} \end{array}$$

for the **G**_2_ of residual power. From (20) the dioptric strength or magnitude of **G**_2_ is

(24) $$\begin{array}{c} \left\|G_{2}\right\|=\left|-2\sin5^{{}^{\circ}}\right|=0.17\;\text{D}. \end{array}$$

Information given is insufficient to determine the principal meridians of the residual power. The magnitude of the resultant cylinder is given by

(25) $$\begin{array}{c} C=2\left(2\right)\sin5^{{}^{\circ}}=0.35\;\text{D}. \end{array}$$

Consequently, advancing the prescribed axis of a 2 D cylinder by 5° produces a residual cylinder power of 0.35 D. Therefore, the wearer's acceptance of a spherocylindrical lens with a rotation in cylinder meridian can be considered in terms of its capacity to blur vision. In this example, the rotation in meridian gives rise to a residual cross-cylinder power of magnitude 0.35/2 D that would not influence the position of the circle of least confusion on the retina.

**Example 2 .**

An eye needs a cylinder correction of −1.00 × 10° but is given −1.50 × 20°. Estimate the residual refraction.

**Figure 5:**
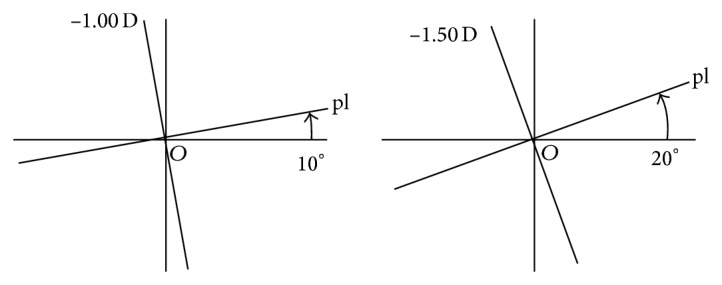
Optical crosses for a prescription lens with principal powers pl along 10° and −1.00 D along 100° and a compensation lens pl along a meridian at 20° and −1.50 D along 110°. In Figure 3(b), these latter data have a power matrix compensation represented at points like *T* where both eigenvectors and eigenvalues differ from those of the prescription power matrix represented at *P*.

*Solution*. The given prescription is *λ* = pl along *α* = 10° and *μ* = −1.00 along *α* + 90° = 100° and the lens supplied is *λ*′ = pl along *α*′ = 20° and *μ*′ = −1.50 along *α*′ + 90° = 110° as in Figure 5. When principal power changes from −1.00 D to −1.50 D in an antistigmatic plane (*P* to *S* in Figure 3(b)), antistigmatic residue is accompanied by a change in scalar power. If only principal meridians change from *α* = 10° to *α*′ = 20° (*P* to *R* in Figure 3(b)), the residual power has zero component of scalar power. The prescription and the compensation may be represented at points like *P* and *T* in Figure 3(b) where both principal powers and their meridians differ. On optical crosses the prescription lens and the lens supplied are as shown in Figure 5.

Equation (6) gives the power **A**_*P*_ for the prescription and **A**_*S*_ for the compensation:

(26) $$\begin{array}{c} A_{P}=-\frac{1}{2}I+\frac{1}{2}\cos20^{{}^{\circ}}J+\frac{1}{2}\sin20^{{}^{\circ}}K,\\ A_{S}=-\frac{3}{4}I+\frac{3}{4}\cos40^{{}^{\circ}}J+\frac{3}{4}\sin40^{{}^{\circ}}K. \end{array}$$

The coefficients of **I** are the semitraces of the prescription and the compensation matrices, and are distinct scalar powers. We determine a matrix power **G** of the residual lens to be added to the power **A**_*S*_ of the compensation to give the power **A**_*P*_ on the prescription:

(27) G=AP−AS=14I+12cos⁡20°−34cos⁡40°J +12sin20°−34sin40°K=14I−0.1047J−0.3111K.

**Figure 6:**
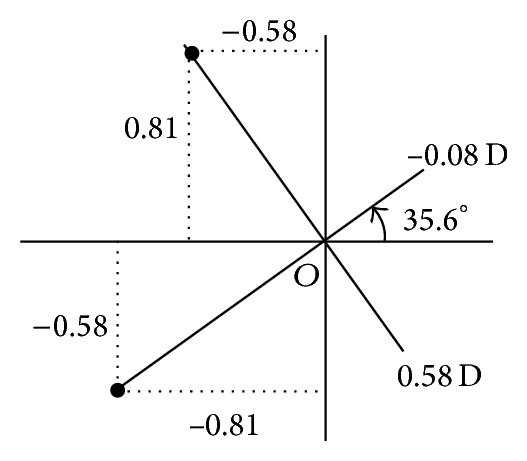
This cross represents the principal meridians and powers of a lens that augment the incorrect meridians and powers of the lens compensation on the right in Figure 5 of Numerical Example 2 to achieve the meridians and powers of the lens prescription on the left.

More digits are retained than are significant. Columns of $Q=\left(\begin{array}{cc} -0.58 & -0.81\\ 0.81 & -0.58 \end{array}\right)$ represent the coordinates of perpendicular meridians sketched in Figure 6 and corresponding entries of $\Lambda =\left(\begin{array}{cc} 0.58 & 0\\ 0 & -0.08 \end{array}\right)\;\text{D}$ represent the principal powers along these meridians that augment the compensation. These are factors of **G** like (3) and (4) are factors of **A** which we have associated with an optical cross like Figure 1. Consider

(28) $$\begin{array}{c} {\left\|G\right\|}^{2}={\left(0.25\right)}^{2}+{\left(-0.1047\right)}^{2}+{\left(-0.3111\right)}^{2}=0.17\;{\text{D}}^{2}. \end{array}$$

Thus the magnitude of **G** is a significant ‖**G**‖ = 0.41 D which is not acceptable. Refer to the prescription and compensation in Figure 5.

This shows that the compensation lens may be augmented approximately by pl 0.5 × 36.

## 4. Discussion

With respect to the prescription, a spectacle lens may have rotated principal meridians along which powers may also vary independently. On a cornea, a toric lens may stabilize away from the prescribed meridian or its lens curvature properties, and hence its power may be inappropriate. In order to make calculations and quantify a lens, these clinical quantities have to be associated with eigenvalues and eigenvectors of a power matrix.

A rotated meridian with no bending of the lens implies that the eigenvectors change while the eigenvalues remain constant. A preferentially curved lens with no meridian rotation implies that the eigenvalues change while the eigenvectors remain independently constant. Deviations from the prescription were the source of the residual refraction as a matrix. Matrix nature and that of the over-refraction in terms of principal powers and meridians were characteristic of the residual refraction in the least processed form (sphere, cylinder, and axis is a processed form).

An antistigmatic segment of a prescription at *P* in Figures 2 and 3(b) is perturbed to yield systematic compensations for an eye. Lens principal powers changed from *P* to those at *S* along their meridians. Residual power (see (14)) is then made up from components of antistigmatic power in the plane of Figure 3(b) that accompanies a scalar (equivalent sphere) power component parallel to the **I**-axis in Figure 2. The nature of the residual refraction was explained for preferential power modification of a toric lens that compensates vision in the spectacle or the corneal plane. We also calculated the strength or magnitude of the residual power that should not exceed an arbitrary tolerance as a general measure of acceptance.

Principal power pairs at *P* in Figure 3(b) are identical to those at *R* but the principal meridians at *R* are different from those at *P*. In (17) the residual contains zero scalar power owing to rotation of principal meridians. Thus an incorrect meridian requires a cross-cylinder lens power only to compensate and obtain the prescription. This residual lens leaves the position of least confusion of rays entering the eye unchanged. We also calculated the strength of this residual cross-cylinder and found that it contained the same arguments as the lens cylinder power.

In Example 1 the residual cross-cylinder power was calculated with (17). Notwithstanding the unknown angles, we were able to calculate the dioptric strength or magnitude of the residual power and hence the magnitude of the resultant cylinder power as the prescribed lens meridian at *P* was advanced to *R* in Figure 3(b). Equation (6) was implemented to write down matrices derived for the principal powers and corresponding meridians at *T* and those at *P*. This numerical problem had a solution containing both antistigmatic and scalar power components of the residual power calculated individually at *R* and *S*. This was confirmed on determining the residual power; a toric lens along new meridians augmented the compensation at *T* and yielded the power of the prescription at *P*.

Compared to the prescription for a toric lens on the cornea or in the spectacle plane, compensation for a patient's eye was not always adequate. Powers were disturbed so that compensation lenses differed in three systematic ways from their prescriptions. Measurements represented on a cross were related to coordinates of basic matrices represented in symmetric dioptric power space. This facilitated the subtraction of relevant matrices to obtain residual powers. The nature of residual powers can be predicted from their position in symmetric dioptric power space. Pythagoras' theorem in diagrams like Figure 2 and matrix scalar products [5] allowed one to associate scalar strengths with residual powers as a tolerance. Eigenvalues and eigenvectors of the residual power were calculated and represented principal powers and meridians as measurements on optical crosses [6].

## References

[1] Guyton D. L. (1977). Prescribing cylinders: the problem of distortion. *Survey of Ophthalmology*.

[2] Harris W. F. (2001). Residual refraction resulting from a rotated spectacle lens. *The South African Optometrist*.

[3] Keating M. P. (1988). *Geometric, Physical and Visual Optics*.

[4] Tjon-Fo-Sang M. J., de Faber J. T., Kingma C., Beekhuis W. H. (2002). Cyclotorsion: a possible cause of residual astigmatism in refractive surgery. *Journal of Cataract and Refractive Surgery*.

[5] Anton H., Rorres C. (2014). *Elementary Linear Algebra: Applications Version*.

[6] Abelman H., Abelman S. (2014). Paraxial ocular measurements and entries in spectral and modal matrices: analogy and application. *Computational and Mathematical Methods in Medicine*.

[7] Harris W. F. (2007). Power vectors versus power matrices, and the mathematical nature of dioptric power. *Optometry and Vision Science*.

[8] Harris W. F. (2003). Inner product spaces of dioptric power and of fundamental and derived properties of optical systems. *South African Optometrist*.

[9] Harris W. F. (1997). Dioptric power: its nature and its representation in three- and four-dimensional space. *Optometry and Vision Science*.

[10] Abelman H., Abelman S. (2009). Bounds and intervals around nonzero cylinder powers in symmetric dioptric power space. *Journal of Biomedical Optics*.

[11] Harris W. F. (2001). Analysis of astigmatism in anterior segment surgery. *Journal of Cataract and Refractive Surgery*.

[12] Abelman H., Abelman S. (2011). Profiles of interval bounds around the coordinates of antistigmatic powers. *Journal of Modern Optics*.

[13] Brown W. L. (2006). Revisions to tolerances in cylinder axis and in progressive addition lens power in ANSI Z80.1-2005. *Optometry*.

